# Cholecystokinin-Mediated RhoGDI Phosphorylation via PKCα Promotes both RhoA and Rac1 Signaling

**DOI:** 10.1371/journal.pone.0066029

**Published:** 2013-06-11

**Authors:** Maria Eugenia Sabbatini, John A. Williams

**Affiliations:** Department of Molecular and Integrative Physiology, University of Michigan, Ann Arbor, Michigan, United States of America; Technische Universität München, Germany

## Abstract

RhoA and Rac1 have been implicated in the mechanism of CCK-induced amylase secretion from pancreatic acini. In all cell types studied to date, inactive Rho GTPases are present in the cytosol bound to the guanine nucleotide dissociation inhibitor RhoGDI. Here, we identified the switch mechanism regulating RhoGDI1-Rho GTPase dissociation and RhoA translocation upon CCK stimulation in pancreatic acini. We found that both Gα13 and PKC, independently, regulate CCK-induced RhoA translocation and that the PKC isoform involved is PKCα. Both RhoGDI1 and RhoGDI3, but not RhoGDI2, are expressed in pancreatic acini. Cytosolic RhoA and Rac1 are associated with RhoGDI1, and CCK-stimulated PKCα activation releases the complex. Overexpression of RhoGDI1, by binding RhoA, inhibits its activation, and thereby, CCK-induced apical amylase secretion. RhoA translocation is also inhibited by RhoGDI1. Inactive Rac1 influences CCK-induced RhoA activation by preventing RhoGDI1 from binding RhoA. By mutational analysis we found that CCK-induced PKCα phosphorylation on RhoGDI1 at Ser96 releases RhoA and Rac1 from RhoGDI1 to facilitate Rho GTPases signaling.

## Introduction

RhoA and Rac1 are small GTP-binding proteins and cycle between two forms: an inactive GDP-bound form and an active GTP-bound form. Several regulatory proteins are implicated in the control of their activity. The guanine nucleotide exchange factors (GEFs) are responsible for their activation by inducing the binding of GTP. Inactivation occurs when the small G protein interacts with a GTPase-activating protein GAP, which hydrolyzes GTP to GDP.

A less studied regulatory protein is the Rho guanine nucleotide dissociation inhibitor (RhoGDI), which not only regulates the activity of Rho GTPases, but also their partitioning between the cytosol and membranes as reviewed in [1]. Because Rho GTPases are post-translationally modified by geranylgeranylation at the C-terminal region [2], which is responsible for targeting Rho GTPases to membranes [3], in the cytosol RhoGDI binds and masks the isoprenyl region. Thus, to allow Rho GTPases to translocate to membranes, the complex needs to dissociate. A number of intracellular signals, including protein kinase C (PKC), calcium, and PKA, have been implicated in the regulation of the dissociation-association cycle of Rho GTPase-RhoGDI complexes. PKCα [4], [5], atypical PKCs [6], [7], p21-activated kinase [8], [9], Src [10], PKA [11], PKG [12] and Ser/Thr kinase Ste20-related kinase (SLK) [13] have been shown to phosphorylate either RhoGDI or Rho GTPases and then induce a dissociation or association of Rho GTPases-RhoGDI complexes.

Three RhoGDI isoforms exist: RhoGDI1, RhoGDI2 and RhoGDI3. Both RhoGDI1 and RhoGDI2 are cytosolic whereas RhoGDI3 is a non-cytosolic isoform which contains a unique amino-terminal extension that targets it to the Golgi complex and other cellular membranes [14]. RhoGDI1 interacts with several members of the Rho family including RhoA, Rac1 and Cdc42; RhoGDI2 similarly associates with the members of Rho family, but with lower affinity. RhoGDI3 interacts predominantly with RhoB and RhoC [1].

Both RhoA and Rac1 have been implicated in the regulation of CCK-induced pancreatic enzyme secretion through an actin cytoskeleton-dependent cellular process [15], [16], [17]. In pancreatic acini, CCK not only increases the amount of GTP-bound forms, but also induces RhoA and Rac1 translocation from the cytosol to membranes [17]. Recently, the heterotrimeric G protein Gα13 has been shown to participate in the activation of RhoA induced by CCK in isolated pancreatic acini [18].

In this study we establish the mechanism regulating RhoA translocation upon CCK stimulation, identify the switch mechanism responsible for RhoGDI1-Rho GTPases dissociation, and study the importance of RhoGDI1 in the response to CCK. Both Gα13 and PKCα, independently, regulate CCK-induced RhoA translocation. Cytosolic RhoA and cytosolic Rac1 are associated with RhoGDI1, and CCK-stimulated PKCα activation releases the complex. By mutational analysis we found that CCK-induced PKCα phosphorylation on RhoGDI1 at Ser96 releases RhoA and Rac1 from RhoGDI1 to facilitate Rho GTPases signaling.

## Materials and Methods

### Materials

Collagenase (CLSPA) was purchased from Worthington Biochemical Co (Lakewood, NJ), bovine albumin fraction V (BSA) was from MP Biomedicals (Solon, OH), H-89, forskolin, 8-Br-cAMP, and soybean trypsin inhibitor (SBTI) were from Sigma Chemical (St. Louis, MO), Dulbecco’s modified Eagle’s medium (DMEM) was from Invitrogen (Carlsbad, CA). The following inhibitors and stimulators were used: sulfated cholecystokinin octapeptide (CCK) was from Research Plus (Bayonne, NJ), A23187, Gö-6976, phorbol 12-myristate 13-acetate (PMA), BAPTA-AM and GF-109203X were from Calbiochem (La Jolla, CA). All other chemical were of reagent grade.

### Antibodies

Antibodies against the following proteins were used: rabbit polyclonal antibody to RhoGDI1 (sc-360), and mouse monoclonal antibody to RhoA (sc-418) from Santa Cruz Biotechnology (Santa Cruz, CA); mouse monoclonal antibody to Rac1(#16118) from Pierce Biotechnology (Rockford, IL); mouse monoclonal antibody to Cdc42 (# 610928) from BD Transduction Laboratories (San Diego, CA); mouse monoclonal antibody to GST (sc-138) from Santa Cruz Biotechnology (Santa Cruz, CA); rabbit polyclonal antibodies to PKCα (#2056), PKCδ (#2058), green fluorescence protein (GFP) (#2555), mouse monoclonal antibodies to β-Gal (#2372), HA-Tag (#2367), Myc-Tag (#2276), rabbit monoclonal antibody to PKCε (#2683) from Cell Signaling Technology (Beverly, MA); mouse monoclonal antibody to β-tubulin from Sigma Chemical (St. Louis, MO).

### Adenoviruses

Adenovirus expressing the RGS domain of p115-RhoGEF, Myc-p115-RGS, was from Patrick J Casey (Duke University, NC) and has been described previously [18], [19]. Adenoviruses encoding dominant-negative PKCα, PKCδ and PKCε were from Dr. J Molkentin (University of Cincinnati, Cincinnati, OH) and have been described previously [20], [21]. Adenoviruses encoding constitutively active (CA)RhoA (RhoA V14), dominant negative (DN)RhoA (RhoA N19), CA-Rac1 (Rac1 V12) and DN-Rac1 (Rac1 N17), each with a triple HA-tag on the N-terminal, have been described previously [16]; viruses expressing β-galactosidase (β-gal) or green fluorescence protein (GFP) were used as a control. Adenoviruses were amplified and purified using ViraBind™ Adenovirus Purification kit from Cell Biolabs Inc (San Diego, CA).

### PKC Peptide Inhibitors

Specific peptide inhibitors of PKCα (αV5-3: Q-L-V-I-A-N), PKCδ (δV1-1: S-F-N-S-Y-E-L-G-S-L), PKCε (εV1-2: E-A-V-S-L-K-P-T) and scrambled peptide (L-S-L-E-L-T-L-K-L-P-A-V) conjugated to a cell permeant 10-amino acid basic peptide from the HIV TAT protein (TAT_47–57_) were obtained from Dr. Daria Mochly-Rosen (Stanford University, School of Medicine, Stanford, CA) and have been described previously [22], [23]. Pancreatic acini were pre-incubated with each PKC inhibitor (1 µM) or Tat in front of a scrambled peptide for 2 h and then stimulated with CCK. The scrambled peptide was used as control.

### Ethics Statement

All the experimental protocols were approved by the University of Michigan Committee on the Use and Care of Animals.

### Isolation of Pancreatic Acini

Mouse pancreatic acini from ICR mice were prepared by enzymatic digestion with collagenase followed by mechanical shearing as previously reported [18]. For overnight incubation, acini were incubated in suspension without shaking at low density in 10 cm Petri dishes in DMEM enriched with 0.1% BSA, 0.01% SBTI and antibiotics, and incubated overnight at 37°C with 5% CO_2_. For the viral infection experiments, 10^7 ^pfu/ml of viruses were added to the culture medium at the beginning of the overnight incubation.

### Determination of RhoA, RhoGDI1, and PKC Isoforms Translocation

Isolated pancreatic acini were stimulated with CCK or other stimulators for 5 min; higher concentrations of CCK are necessary to stimulate overnight incubated acini due to a loss of sensitivity. Translocation assay was determined as previously described [17]. The pellet obtained from Beckman Optima TLX ultracentrifuge was resuspended in 250 µl of lysis buffer containing 1% Triton X-100 and collected as the particulate fraction. The amount of RhoA, RhoGDI1 and PKC isoforms was determined by immunoblot analysis. In another group of experiments, the soluble fraction was generated for the co-immunoprecipitation. The quantitative analysis of translocation was performed using density analysis software from FluorChem® Imaging Systems (Alpha Innotech). Images were taken using Alpha Innotech FluorChem™ 8000 imaging system (Alpha Innotech, San Leandro, CA, USA) for densitometry analysis with AlphaChem™ imaging software (Alpha Innotech). The density of each band was measured using AlphaEaseFC™ software and then, each result was normalized to its membrane control β-tubulin by dividing the integrated density volume (IDV) by the IDV of β-tubulin to account for differences in loaded amounts.

### Determination of RhoA and Rac1 Activation

RhoA and Rac1 activation were determined as previously described [18]. Rhotekin-RBD protein GST-beads (#RT02) (100 µg; 30 µl) or PAK-GST Protein Beads (#PAK02) (20 µg; 20 µl) (Cytoskeleton Inc, Denver, CO) were used to bind the active GTP-bound form of RhoA or Rac1, respectively.

### Affinity Precipitation using Glutathione S-transferase (GST)-fusion Proteins

Plasmids of GST-constitutively active RhoA (pGEX-4T3-RhoA-Q63L), GST-dominant negative RhoA (pGEX-2T-RhoA-T19N), GST-constitutively active Rac1 (pGEX-2T-Rac1-Q61L), and GST-dominant negative Rac1 (pGEX-2T-Rac1-T17N) were from Addgene (Cambridge, MA). GST-empty vector was used as control. All were expressed in Escherichia coli by induction with 0.1 mM isopropyl β-D-thiogalactopyranoside for 8 h at room temperature and purified from bacterial lysates with glutathione-Sepharose 4B beads (GE Healthcare, Waukesha, WI). Isolated pancreatic acini were homogenized with lysis buffer containing 20 mM HEPES, pH 7.5, 100 mM sodium chloride, 5 mM magnesium chloride, 1 mM dithiothreitol, 1% Triton X-100, 1 mM PMSF, 10 mM sodium fluoride, 10 µg/ml aprotinin, 10 µg/ml leupeptin, 1 mM sodium vanadate. Lysates were centrifuged at 14,000×g for 10 min at 4°C. The supernatant (2000–2500 µg of proteins) was first precleared with 50 µg of glutathione sepharose beads for 30 min at 4°C, followed by 1 h rotation at 4°C with 50 µg of purified GST-fusion proteins immobilized on glutathione-Sepharose beads. Beads were washed 3 times with lysis buffer and eluted for 30 min at room temperature using buffer containing 75 mM HEPES, pH 8, 100 mM sodium chloride, 10 mM reduced glutathione, 20 mM EDTA, 0.1% Triton X-100, 5 mM dithiothreitol, 1 mM PMSF, 10 mM sodium fluoride, 10 µg/ml aprotinin, 10 µg/ml leupeptin, 1 mM sodium vanadate. The eluates were subjected to Western-blotting for immunodetection of RhoGDI1.

### Co-Immunoprecipitation

After stimulation with CCK, pancreatic acini were collected by centrifugation, washed once with phosphate buffered saline (PBS) and lysed with immunoprecipitation buffer (400 µl) containing 50 mM TRIS HCl, pH 7.5, 150 mM sodium chloride, 5 mM EDTA, 10 mM sodium pyrophosphate, 25 mM β-glycerolphosphate, 0.1% Triton X-100, 1 mM PMSF, 10 µg/ml leupeptin, 10 µg/ml aprotinin, 1 mM sodium vanadate, 25 mM sodium fluoride, 1 mM dithiothreitol. Total lysate (2500 µg) or cytosolic fraction (1000 µg), obtained as explained above, was rotated overnight with polyclonal rabbit anti-RhoGDI1 antibody (2 µg) at 4°C followed by complex collection with protein A-agarose immunoprecipitation reagent (sc-2001, Santa Cruz Biotechnology, Inc) for 1 h at 4°C. The immunocomplexes were washed three times with lysis buffer and processed for Western-blotting analysis as described previously [18]. In another experiment, acini expressing (CA)RhoA, (DN)RhoA, (CA)-Rac1 or (DN)-Rac1, each with a triple HA-tag on the N-terminal, were lysed and immunoprecipitated using monoclonal anti-HA agarose conjugate clone HA-7 (50 µl) (Sigma Chemical, St. Louis, MO). After 1 h rotation at 4°C, the resin was washed four times using RIPA buffer (1% sodium deoxycholate, 0.1% SDS, 1% Triton X-100, 0.01 M Tris-HCl, pH 8, 0.14 M sodium chloride), and then the immunoprecipitated HA-tagged proteins were specifically eluted with HA peptide (100 µg/ml) (Sigma Chemical, St. Louis, MO). The quantitative analysis of co-immunoprecipitation was performed using density analysis software from FluorChem® Imaging Systems (Alpha Innotech). The density of each band was measured using AlphaEaseFC™ software and then, each result was normalized to the amount of RhoGDI by dividing the integrated density volume (IDV) by the IDV of RhoGDI to account for differences in loaded amounts. The dissociation levels were normalized to control  = 1.0 in each assay and expressed as fold change.

### Detection of the Expression of RhoGDI Isoforms in Mouse Pancreatic Acini

The expression of three different isoforms of RhoGDI in mouse pancreatic acini was assessed by reverse transcriptase-polymerase chain reaction (RT-PCR). Total RNA was isolated from brain, whole pancreas and isolated pancreatic acini using TRIzol reagent (Invitrogen) to synthesize first-strand cDNAs with TaqMan RT-PCR kit (Applied Biosystems, Branchburg, NJ). Intactness of RNA was assessed by OD 260/280 and agarose gel electrophoresis. One µg of cDNA was used in each PCR reaction. Amplification with Taq DNA Polymerase from Expand High Fidelity Enzyme Mix kit (Roche Diagnostics, IN) was conducted using specific primers: mouse RhoGDI1 (382 bp) (Genebank accession NM_133796): 5′- GGAGATCCAGGAACTGGACA-3′ (sense), 5′- CTCCATGGGTGTCAGGAACT-3′ (antisense); mouse RhoGDI2 (323 bp) (Genebank accession NM_007486): 5′- CAGGTGAGGTGGTCCTGTTT-3′ (sense), 5′- CCCATCACCATGGACCTTAC-3′ (antisense), and mouse RhoGDI3 (344 bp) (Genebank accession NM_008113): 5′- AAGAAGAGCATGCTGGCAAT-3′ (sense), 5′- ACCATGAAGATGGCCTTGTC-3′ (antisense) (Invitrogen, Carlsbad, CA). The primers were designed with Invitrogen Oligoperfect Designer based on gene sequences obtained from the GenBank NCBI Sequence Viewer (http://www.ncbi.nlm.nih.gov).

The presence of RhoGDI1 protein was evaluated by Western-blotting using a rabbit polyclonal antibody from Santa Cruz Biotechnology (sc-360).

### Immunolocalization of RhoGDI1

For immunolocalization of RhoGDI1, freshly isolated pancreatic acini were stimulated with CCK (300 pM) for 15 min at 37°C. Cryostat sections (6 µm thick) were made as previously described [18] and mounted on SuperFrost Plus slices (Fischer) and incubated with PBS containing 2% normal goat serum and 0.3% Triton X-100 followed by a rabbit polyclonal antibody to RhoGDI1 (diluted 1∶2000). The sections were washed with PBS and incubated with 1∶500 dilutions of goat anti-rabbit secondary antibody conjugated to Alexa 594. Prolong Gold with 4, 6-diamino-2-phenylindole (DAPI) was added to mounting medium to counterstain nuclei. Fluorescence images were captured with Olympus 500 Fluoview Confocal microscope using a x60(1.40 NA) oil immersion objective, and processed with Adobe Photoshop software.

### Construction of Recombinant RhoGDI1 Adenoviruses

Plasmid of wild-type (WT) human RhoGDI1 cloned into pcDNA3.1+ was from cDNA Resource Center, Missouri University of Science and Technology. The S34A-RhoGDI1, S96A-RhoGDI1, S34D-RhoGDI1 and S96D-RhoGDI1 mutants were made using the QuikChange® II XL Site-Directed Mutagenesis Kit from Stratagene (#200521, Cedar Creek, TX). Each mutation was confirmed by sequencing using T7 primers. Adenoviral delivery plasmids were constructed by insertion of the annealed WT or mutant RhoGDI1 into the 5′KpnI and 5′XhoI sites of the pAdTrack-CMV vector (Stratagene, Cedar Creek, TX) and then into the pAdEasy vector according to the method of He et al. [24].

### Measurement of Amylase Secretion

Amylase secretion was determined as previously reported [18]. Results were expressed as a percentage of initial acinar amylase content.

### Statistical Analysis

Results were expressed as means ± SEM of 3 to 6 separate experiments. The statistical analysis was performed by ANOVA following by the Student-Newman-Keuls test. A p of 0.05 or less was considered statistically significant.

## Results

### PKCα is Involved in CCK-induced RhoA Translocation

**Figure 1:**
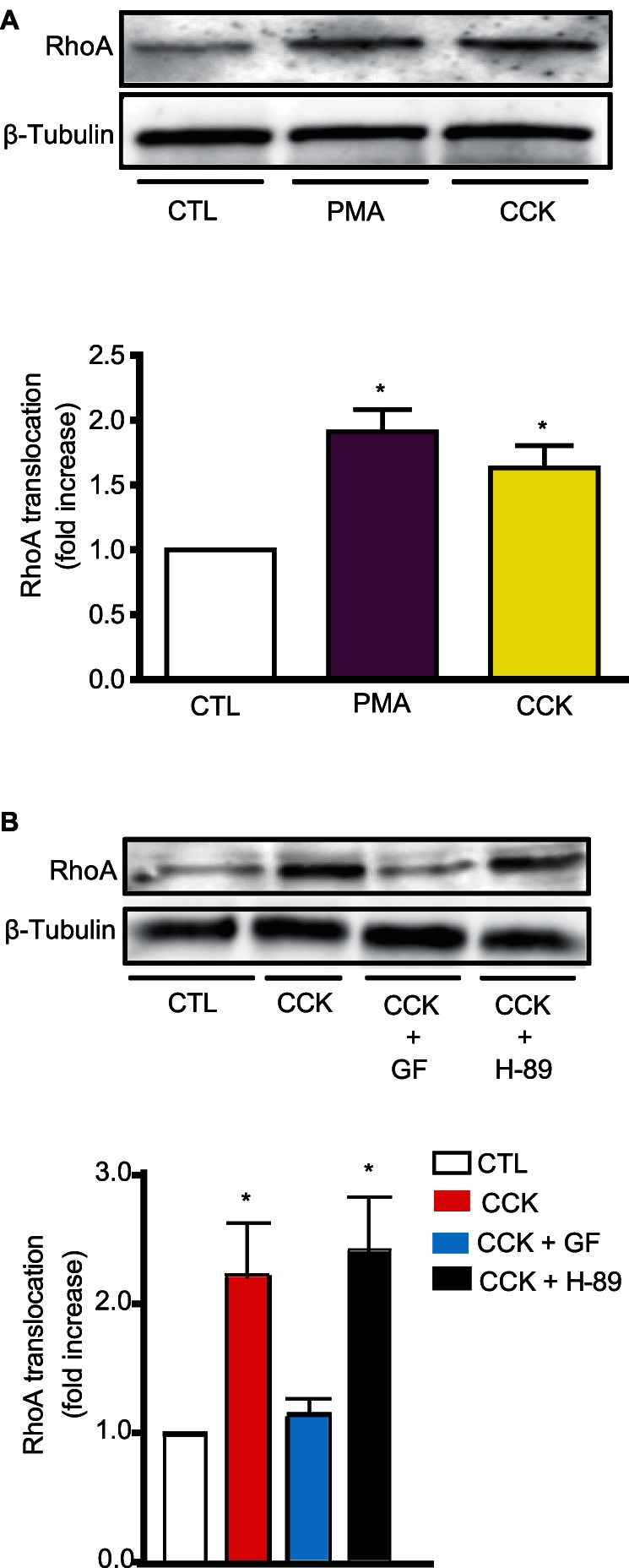
PKC is required for CCK-induced RhoA translocation. **A)** Freshly isolated pancreatic acini were stimulated with PMA (500 nM) or CCK (300 pM) for 5 min and RhoA translocation determined. **B)** Acini were pretreated with the PKC inhibitor GF-109203X (GF, 5 µM) or the PKA inhibitor H-89 (10 µM) for 30 min and then stimulated with 300 pM CCK for 5 min. RhoA translocation was determined. For both **A)** and **B)** a representative immunoblot for RhoA of acinar particulate fraction and a quantitative analysis of RhoA translocation are shown. Equivalent loading was confirmed using β-tubulin. All values shown are means ± SE (n  = 4 experiments). * p<0.05 vs. control (CTL).

Pathways involved in the secretory response to CCK include the phospholipase C (PLC)/diacylglycerol/PKC, PLC/IP3/calcium and cAMP/PKA pathways. We found that PKC is involved in CCK-induced RhoA translocation because the phorbol ester PMA increased RhoA translocation to the same extent as CCK **(** **Fig. 1A** **).** When PKC activity was inhibited by GF-109203X, CCK-induced translocation was inhibited whereas when PKA activity was inhibited by H-89, CCK-induced RhoA translocation was not modified **(** **Fig. 1B** **).** In addition, the presence of agonists which induce an increase in cAMP levels did not induce RhoA translocation (**Fig. S1A**). Intracellular calcium was not required for CCK-induced RhoA translocation because the presence of a calcium chelator, BAPTA-AM, did not modify the response to CCK (**Fig. S1B**).

**Figure 2:**
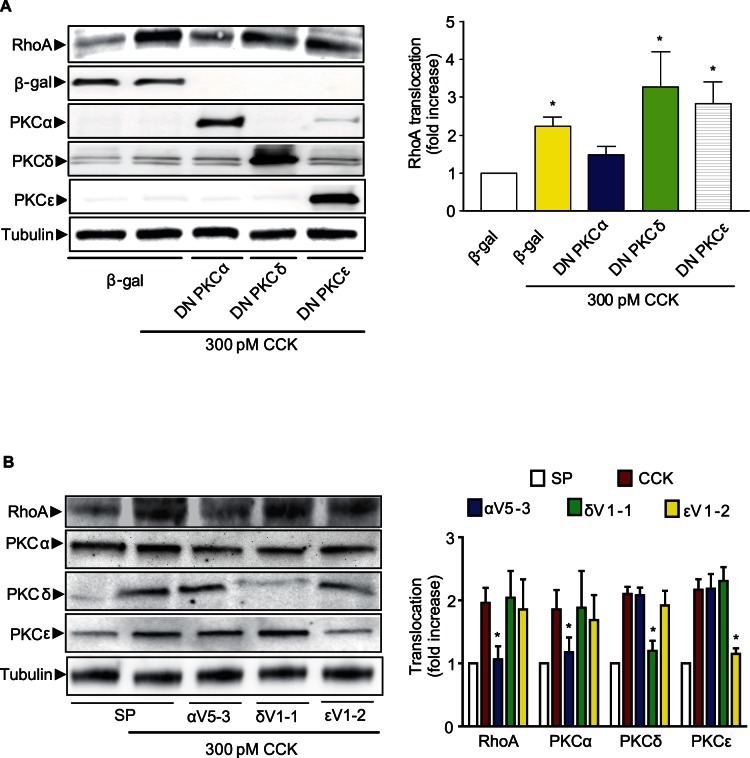
PKCα is the isoform required for CCK-induced RhoA translocation. **A)** β-Gal, DN-PKCα, DN-PKCδ, and DN-PKCε were expressed in isolated pancreatic acini by means of recombinant adenoviruses with overnight incubation. Acini were stimulated with CCK (300 pM) for 5 min and RhoA translocation to the particulate fraction was evaluated. The expression of β-Gal and each DN-PKC isoform was confirmed by Western-blotting. **B)** Freshly pancreatic acini were incubated for 2 h with PKC peptide inhibitors of PKCα (αV5-3, 1 µM), PKCδ (δV1-1, 1 µM), PKCε (εV1-2, 1 µM) or scrambled peptide (SP) (1 µM) and then stimulated with CCK (300 pM) for 5 min. Western-blotting analysis of soluble and particulate fractions from isolated pancreatic acini was carried out to demonstrate isozyme-selective inhibition. For both **A)** and **B)**: The left panel shows a representative immunoblot for RhoA. Only PKCα is required for CCK-stimulated RhoA translocation. The right panel shows a quantitative analysis of translocation. Equivalent loading was confirmed using β-tubulin. Values in both **A)** and **B)** are means ± SE (n  = 4 experiments). * p<0.05 vs. control or scrambled peptide.

Multiple PKC isoforms have been described and at least three isoforms are activated by CCK in pancreatic acini: PKCα, PKCδ and PKCε [21], [25], [26]. Although PKCζ is also present in pancreatic acini, PKCζ has not been involved in the response to CCK [21], [27]. To examine which PKC isoform mediates CCK-induced RhoA translocation, dominant-negative PKC isoform mutants were expressed in mouse pancreatic acini using an adenoviral delivery. Only the expression of dominant negative PKCα mutant inhibited CCK-induced RhoA translocation (**Fig. 2A**).

To confirm these results by another approach, we performed a pharmacological analysis using PKC isoform specific peptide inhibitors. The pre-treatment with PKCα-selective peptide inhibitor, αV5-3, essentially inhibited CCK-induced RhoA translocation whereas other PKC peptide inhibitors did not modify the response to CCK (**Fig. 2B**). The specificity of each peptide inhibitor was confirmed by the observation that the translocation of each PKC isoform induced by CCK was blocked by its specific peptide inhibitor (**Fig. 2B**).

**Figure 3:**
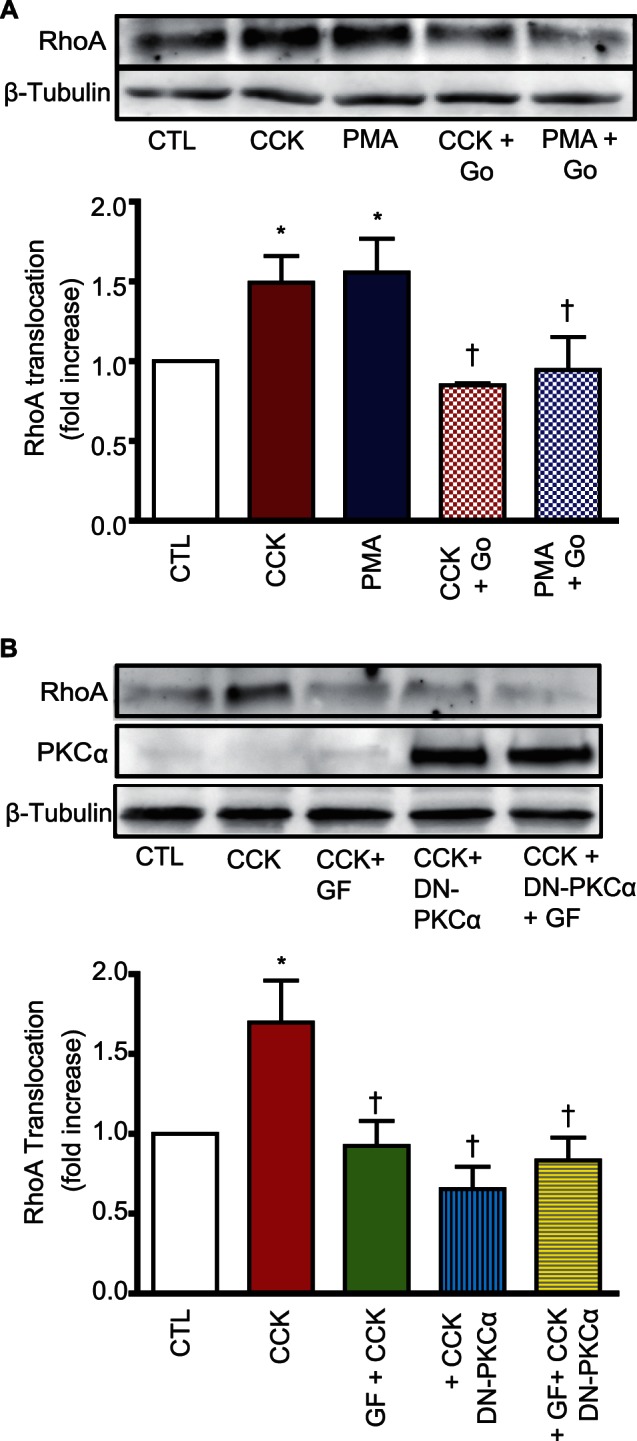
No additional conventional PKC isoform is involved in the response to CCK. **A)** Freshly isolated pancreatic acini were pre-treated with the conventional PKC isoform inhibitor Gö6976 (Go, 2 µM) and stimulated with PMA (500 nM) or CCK (300 pM) for 5 min. RhoA translocation was determined. **B)** Pancreatic acini expressing DN-PKCα or β-gal (control) were pre-treated with the broad spectrum PKC inhibitor GF-109203X (GF, 5 µM). Acini were stimulated with CCK (300 pM) for 5 min and then RhoA translocation was evaluated. For both **A)** and **B)** a representative immunoblot for RhoA of acinar particulate fraction and a quantitative analysis of RhoA translocation are shown. Equivalent loading was confirmed using β-tubulin. The expression of DN-PKCα was confirmed by Western-blotting of the PKCα. All values shown are means ± SE (n  = 5 experiments). * p<0.05 vs. control (CTL), †: p<0.05 vs CCK or PMA.

Pancreatic acini were also pre-incubated with the conventional PKC isoform inhibitor Gö6976 (2 µM) and stimulated with CCK (300 pM) or PMA (500 nM). The response to both agonists was abolished in the presence of Gö6976 **(** **Fig. 3A** **)**. These results further support the participation of PKCα in RhoA translocation induced by CCK. To test for further inhibition related to a different conventional PKC isoform, pancreatic acini expressing DN-PKCα or β-Gal, were pre-incubated with or without GF-109203X (the broad spectrum PKC inhibitor). Pancreatic acini were stimulated with CCK (300 pM) and RhoA translocation was analyzed. The results showed that there is no further inhibition in the response to CCK; all the treatment abolished RhoA translocation as the same extent **(** **Fig. 3B** **)**, which indicates that there is no other conventional PKC isoform involved.

### Gα13 is also Responsible for CCK-induced RhoA Translocation

**Figure 4:**
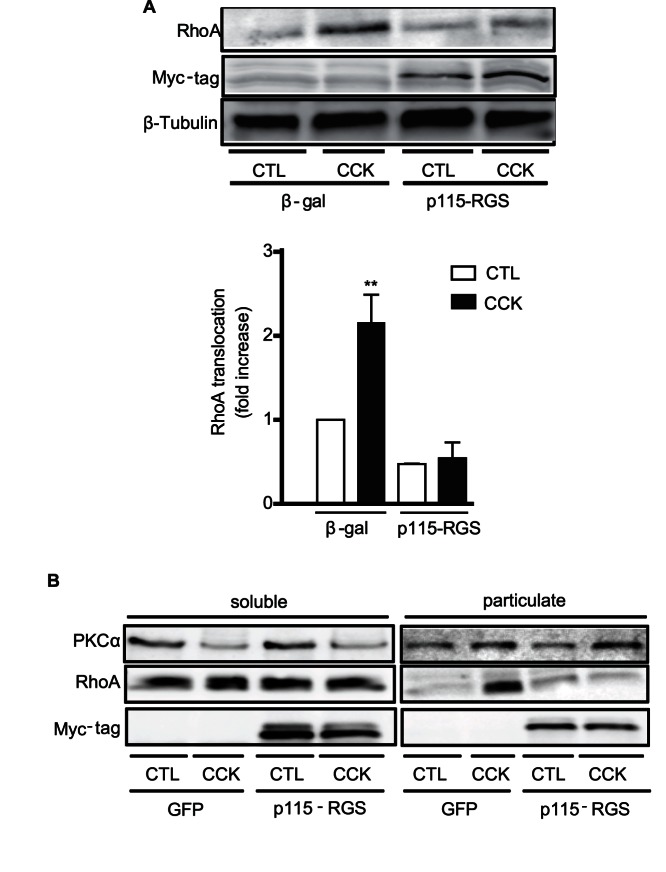
Gα13 is involved in CCK-induced RhoA translocation in a PKCα-independent manner . **A)** Overnight incubated pancreatic acini expressing p115-RGS or β-Gal (control) were stimulated with or without 300 pM CCK for 5 min and RhoA translocation was determined. A representative immunoblot shows that the expression of p115-RGS inhibits CCK-induced RhoA translocation. The expression of p115-RGS was confirmed by Western-blotting of the Myc-tag. Equivalent loading was confirmed using β-tubulin. *Bottom:* quantitative analysis of RhoA translocation. Values are means ± SE (n  = 4 experiments). ** p<0.01 vs. control. **B)** Overnight incubated pancreatic acini expressing Myc-p115-RGS were stimulated with 300 pM CCK for 5 min and then the translocation of both PKCα and RhoA was evaluated. Representative immunoblots for PKCα and RhoA show that Gα13 is not required for the translocation of PKCα induced by CCK, though it is necessary for the translocation of RhoA induced by CCK. The expression of p115-RGS was confirmed by Western-blotting of the Myc-tag.

We have recently shown that Gα13 mediates CCK-induced RhoA activation in mouse pancreatic acini [18]. To study whether Gα13 is also involved in CCK-induced RhoA translocation, acini were infected with adenoviruses encoding the RGS like domain of p115-RhoGEF, p115-RGS, which selectively binds and inhibits Gα13 [19], [28]. In control acini CCK induced translocation of soluble RhoA to the particulate fraction, while in pancreatic acini expressing p115-RGS, CCK was unable to induce RhoA translocation **(** **Fig. 4A** **)**. These results indicate that both Gα13 and PKCα are required for CCK-induced RhoA translocation.

### PKCα and Gα13 have Independent Mechanisms to Induce RhoA Translocation

Because both PKCα and Gα13 are activated by CCK [18], [21] and involved in RhoA translocation, we studied whether Gα13 activates PKCα upon CCK stimulation or whether they are independent pathways. As previously shown in control acini [21], CCK induced PKCα translocation from the soluble to the particulate fraction. The expression of p115-RGS in incubated pancreatic acini did not modify CCK-induced PKCα translocation (**Fig. 4B**), indicating that although both Gα13 and PKCα induce RhoA translocation, it is likely that they are acting through different pathways.

### Both RhoGDI1 and RhoGDI3 are Expressed in Pancreatic Acini

Because in several cell types Rho GTPases are predominantly associated with RhoGDIs, the expression of three known RhoGDI isoforms, RhoGDI1, RhoGDI2, RhoGDI3 was studied. Using RT-PCR we found that in isolated pancreatic acini two isoforms were expressed: RhoGDI1 and RhoGDI3 (**Fig. S2A**). The expression of RhoGDI3 in the whole pancreas has previously been shown [29]. Because both RhoA and Rac1 are preferentially associated with RhoGDI1 [1], this isoform was further studied. We also found that in pancreatic acini RhoGDI1 was highly localized in the cytosol in both non-stimulated and stimulated conditions (**Fig. S2B**). To confirm the Western-blot results, we performed an immunohistochemical study with confocal microscopy using antibody against RhoGDI1. Endogenous RhoGDI1 was diffusely localized in the cytosol; upon CCK stimulation there were no changes in overall RhoGDI1 localization at any studied time point (5 min and 15 min) **(Fig. S2C,** only 15 min is shown**)**.

### Both RhoA and Rac1 are Associated with RhoGDI1 in the Cytosol and upon CCK Stimulation the Complexs are Dissociated

**Figure 5:**
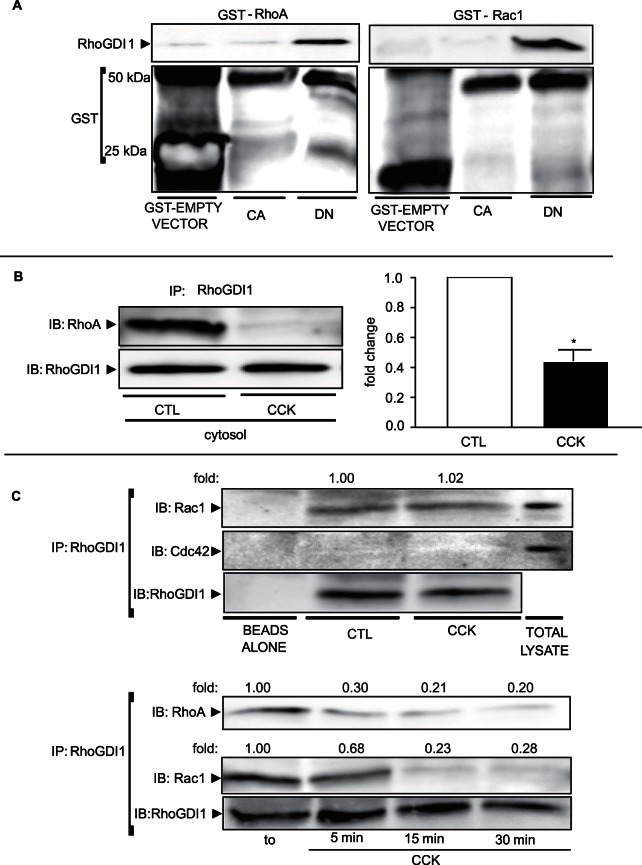
RhoGDI1 interacts with inactive RhoA and Rac1 and upon CCK stimulation both complexes are dissociated. **A)** Total lysates were incubated with GST-CA-RhoA, GST-DN-RhoA, GST-CA-Rac1 and GST-DN-Rac1. GST-empty vector was used as a control. The beads were eluted with elution buffer containing reduced glutathione, and then, total lysates, as well as elutes were subjected to Western-blotting for RhoGDI1. A representative immunoblot for RhoGDI1 shows that only GST-DN-RhoA and GST-DN-Rac1 are able to interact with RhoGDI1. Comparable amount of GST-fusion proteins was shown by Western-blot using anti-GST antibody. **B)** Isolated pancreatic acini were stimulated with 300 pM CCK for 5 min. A cytosolic fraction was obtained using differential centrifugation and co-immunoprecipitated with anti-RhoGDI1. *Left:* representative immunoblots for RhoA and RhoGDI1 shows that upon CCK stimulation, RhoA is released from RhoGDI1. Comparable amount of immunoprecitated RhoGDI1 was confirmed by Western-blotting using anti-RhoGDI1 antibody. *Right:* quantitative analysis of RhoGDI1 associated with RhoA. Data are representative of 4 independent experiments; the dissociation levels were normalized to control  = 1.0 in each assay and expressed as fold change.Values are means ± SE. *: p<0.05 vs control. **C)** *Top*: Acini were stimulated with CCK (300 pM) for 5 min. A representative immunoblot for Rac1 and Cdc42 shows Rac1, but not Cdc42, is associated with RhoGDI1. *Bottom*: Acini were stimulated with CCK (300 pM) for 5, 15 or 30 min. A representative immunoblot for RhoA and Rac1 shows that RhoA is dissociated from RhoGDI1 faster than Rac1 (5 min vs 15 min). Comparable amount of immunoprecitated RhoGDI1 was confirmed by Western-blotting using anti-RhoGDI1 antibody. Data are representative of 3 independent experiments; the dissociation levels were normalized to control  = 1.0 in each assay and expressed as fold change.

To determine whether Rho GTPases are able to interact with RhoGDI1 in pancreatic acini and how this is affected by nucleotide binding state, two approaches were used: affinity pull-down assay and co-immunoprecipitation. Endogenous RhoGDI1 from total lysates strongly associated with glutathione beads conjugated with both recombinant GST-DN-RhoA and GST-DN-Rac1, but not with GST-CA-proteins or control beads (GST-empty vector) (**Fig. 5A**). In another experiment, HA-CA-proteins or HA-DN-proteins were expressed in pancreatic acini and then HA-immunoprecipitation was carried out. We found that endogenous RhoGDI1 is associated with HA-DN-proteins, but not with HA-CA-proteins (**Fig. S3**). We also shown that RhoA and RhoGDI1 could be co-immunoprecipitated from the cytosolic fraction and after 5 min stimulation with CCK, the interaction between RhoGDI1 and RhoA was reduced to 42±5% of control (**Fig. 5B**). Rac1, but not Cdc42, could be also co-immunoprecipitated with RhoGDI1 from the cytosolic fraction **(** **Fig. 5C** **).** However, upon CCK stimulation, Rac1 was dissociated from RhoGDI1 later than RhoA: whereas RhoA was dramatically dissociated at 5 min, Rac1 was dramatically dissociated at 15 min and both proteins stayed dissociated from RhoGDI1 for more than 30 min (**Fig. 5C**). A slight decrease in the association between Rac1 and RhoGDI was seen at 5 min. These findings indicate that CCK stimulation leads to the dissociation of complexes between either RhoA or Rac1 and cytosolic RhoGDI1.

### PKCα, but not Calcium, is Required for the Dissociation of RhoGDI1-RhoA Complex

**Figure 6:**
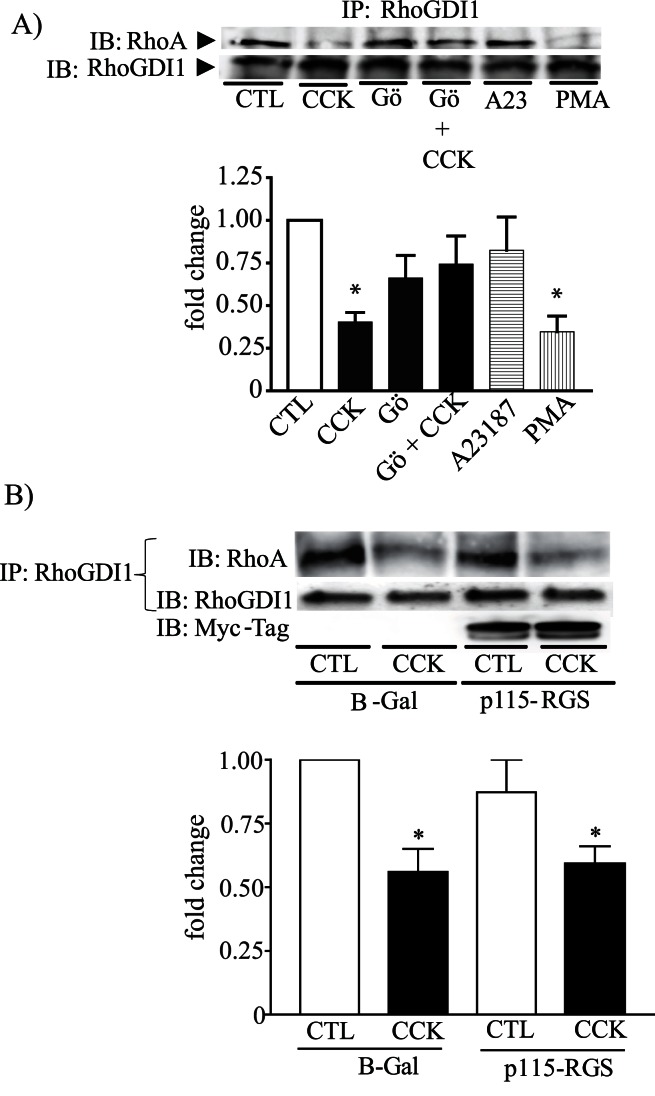
PKCα, but not Gα13, promotes the release of RhoA from RhoGDI1. (**A**) Isolated pancreatic acini were pretreated with the PKCα inhibitor Gö6976 for 30 min and then stimulated with 300 pM CCK for 5 min. Another group of acini were stimulated with either 2 µM A-23187 (A23) or 500 nM PMA. (**B**) Overnight incubated pancreatic acini expressing p115-RGS or β-Gal (control) were stimulated with or without 300 pM CCK for 5 min. In both cases, acini were lysed and immunoprecipitated with rabbit anti-RhoGDI1. **A, B)** *Top:* A representative immunoblot for RhoA shows that the PKCα, but not Gα13, is requires for RhoA-RhoGDI1 complex dissociation. Comparable amount of immunoprecitated RhoGDI1 was confirmed by Western-blotting using anti-RhoGDI1 antibody. *Bottom:* A quantitative analysis of RhoGDI1 associated with RhoA. Values are means ± SE (n  = 4 experiments). *: p<0.05 vs control.

To determine which second messenger is able to dissociate the RhoA-RhoGDI1 complex upon CCK stimulation, several inhibitors and stimulators were used. PMA, similar to CCK, decreased the association of RhoA-RhoGDI1 by 63% (to 37±7% of control, p<0.05) while increasing calcium by A23187 had no effect **(** **Fig. 6A** **)**. In the presence of the conventional PKC inhibitor Gö6976, which inhibits PKCα, but not PKCδ, PKCε or PKCζ, a decrease in the dissociation of RhoA from RhoGDI1 in response to CCK was observed (**Fig. 6A**). Gö6976 also prevented the dissociation of RhoA from RhoGDI1 induced by PMA **(data not shown).** These results indicate that PKCα, upon CCK stimulation, is responsible for the RhoGDI1-RhoA dissociation. Gα13 did not appear to be involved in the dissociation of the complex because the expression of p115-RGS did not modify the response to CCK **(** **Fig. 6B** **)**.

### Phosphorylation at Ser96 in RhoGDI1 is Required for the Dissociation of Complex and Rho GTPases Activation

**Figure 7:**
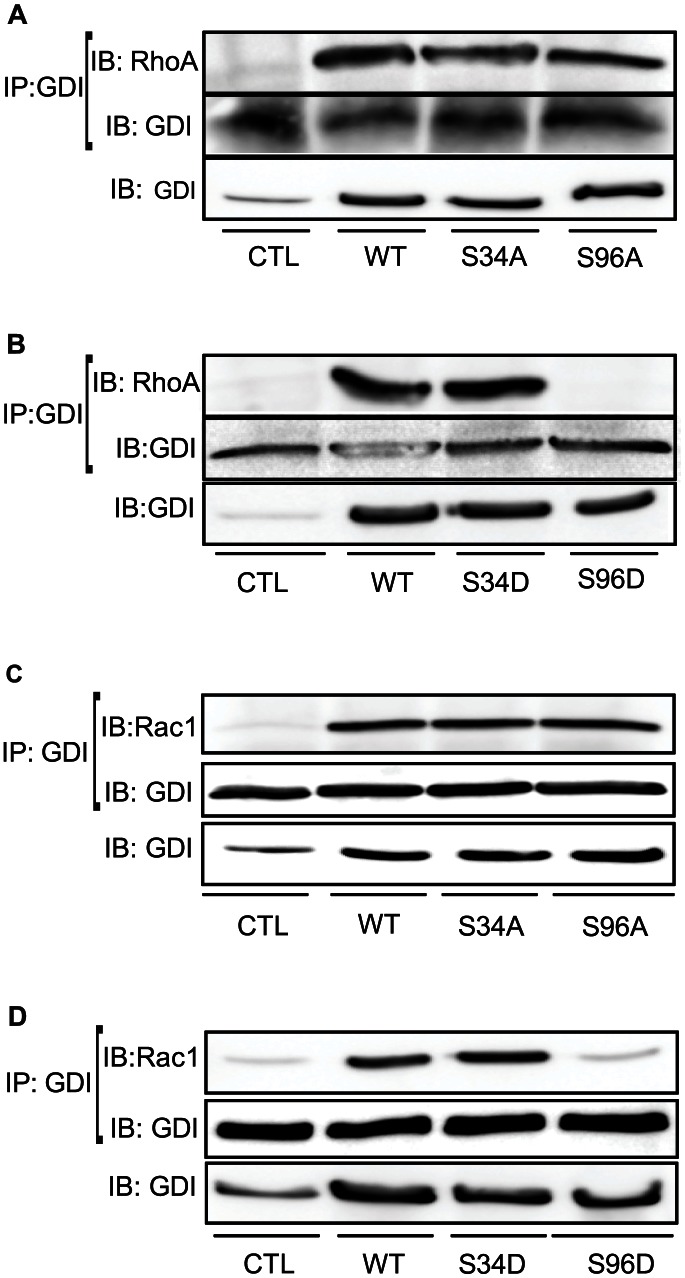
Pancreatic acini overexpressing mutant S96D-RhoGDI1 exhibit a RhoA and Rac1-binding deficiency. Wild-type-RhoGDI1 (WT), the phosphodefective mutants S34A-RhoGDI1 and S96A-RhoGDI1, as well as the phosphomimetic mutants S34D-RhoGDI1 and S96D-RhoGDI1 were expressed in pancreatic acini using adenoviral delivery. The association between either RhoA (**A, B**) or Rac1 (**C, D**) with RhoGDI1 was studied in total lysates using co-immunoprecipitation. Comparable expression of the WT-RhoGDI1, as well as mutants was analyzed by Western-blotting using anti-RhoGDI1 antibody. A representative immunoblot shows that only in acini expressing the phosphomimetic mutant S96D-RhoGDI1, the complex formation was inhibited. Comparable amount of immunoprecitated RhoGDI1 was confirmed by Western-blotting using anti-RhoGDI1 antibody (n  = 4 experiments).

**Figure 8:**
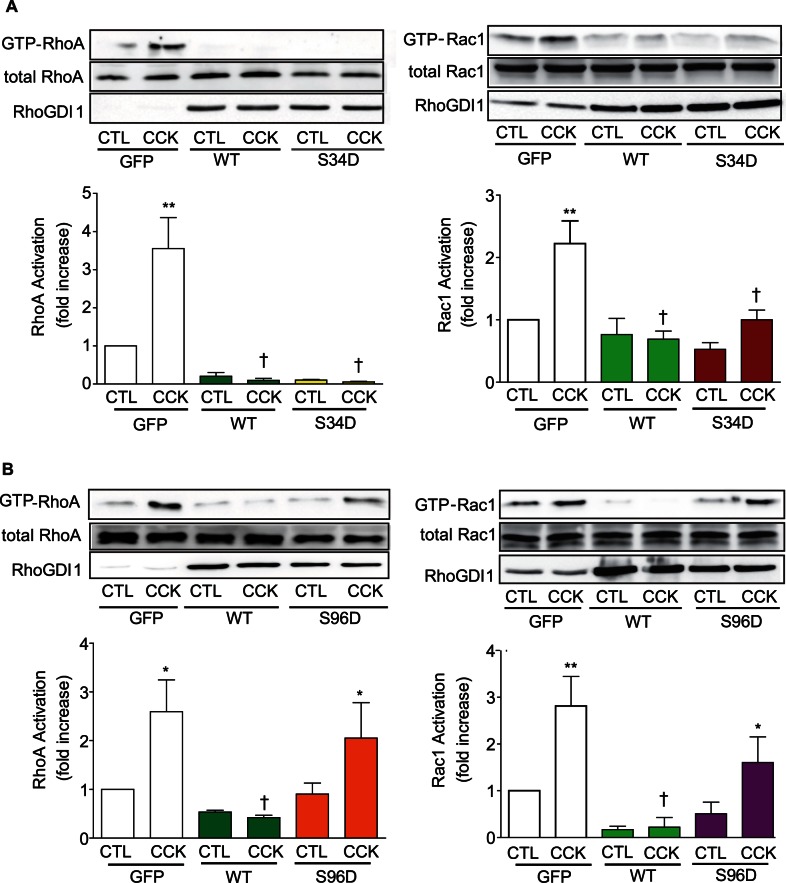
Phosphorylation at Ser96 in RhoGDI1 is required for CCK-induced RhoA and Rac1 activation. WT-RhoGDI1 and the phosmomimetic mutants S34D-RhoGDI and S96D-RhoGDI were expressed in pancreatic acini using adenoviral delivery. The expression of GFP was used as a control. Overnight incubated acini were stimulated with CCK (300 pM) for 5 min and then RhoA activation or 10 min and then Rac1 activation were determined using a pull-down assay. *Top*: A representative immunoblot shows that the expression of WT-RhoGDI1 inhibits CCK-induced RhoA activation or Rac1 activation. Only the expression of phosphomimetic mutant S96D-RhoGDI1 induced CCK-stimulated GTP-RhoA and GTP-Rac1 levels (Representative of 4 experiments). Comparable expression of WT-RhoGDI1, as well as mutants was analysed by Western-blotting using an anti-RhoGDI1 antibody. *Bottom:* Quatitative analysis of either RhoA or Rac1 activation. Values are means ± SE (n  = 4 experiments). *: p<0.05 vs control and †: p<0.05 vs CCK.

Two PKCα phosphorylation sites on RhoGDI1 have been identified: Ser34 [5] and Ser96 [4]. Because in pancreatic acini PKCα was involved in the dissociation of RhoA from RhoGDI1, mutations at Ser34 or Ser96 in RhoGDI1 were carried out; serines were replaced with either alanine (A) to obtain the phosphodefective mutants or with aspartic acid (D) to obtain the phosphomimetic mutants. In pancreatic acini overexpressing WT-RhoGDI1 there was an increase in the association between either RhoA or Rac1 and RhoGDI1 compared with control acini (**Fig. 7A-D**) and an inhibition of CCK-induced RhoA or Rac1 activation **(** **Fig. 8A and 8B** **, Fig. S4A and S4B)**. CCK-induced RhoA translocation was also reduced in acini expressing WT-RhoGDI1 (**Fig. S5A–D**). The expression of the phosphodefective mutants S34A-RhoGDI1 and S96A-RhoGDI1 also increased the association of RhoGDI1 with either RhoA or Rac1 (**Fig. 7A and 7C**) and inhibited CCK-stimulated RhoA activation **(Fig. S4A and S4B)** and RhoA translocation (**Fig. S5C and S5D**). By contrast, the expression of the phosphomimetic mutant S96D-RhoGDI1 prevented the association (**Fig. 7B and 7D**), and did not modify CCK-stimulated RhoA or Rac1 activation (**Fig. 8B**), as well as CCK-induced RhoA translocation (**Fig. S5B**), whereas the expression of S34D-RhoGDI1 acted similarly as WT-RhoGDI1 (**Fig. 7B, 7D** **,** **8A** **and Fig. S5A**). These results indicate that the phosphorylation of native Ser96 in RhoGDI1 or its replacement with aspartic acid to mimic the phosphorylation state is required for releasing RhoA and Rac1 and their signaling.

### Inactive Rac1 Prevents the Inhibitory Effect of RhoGDI1 on RhoA Activation

**Figure 9:**
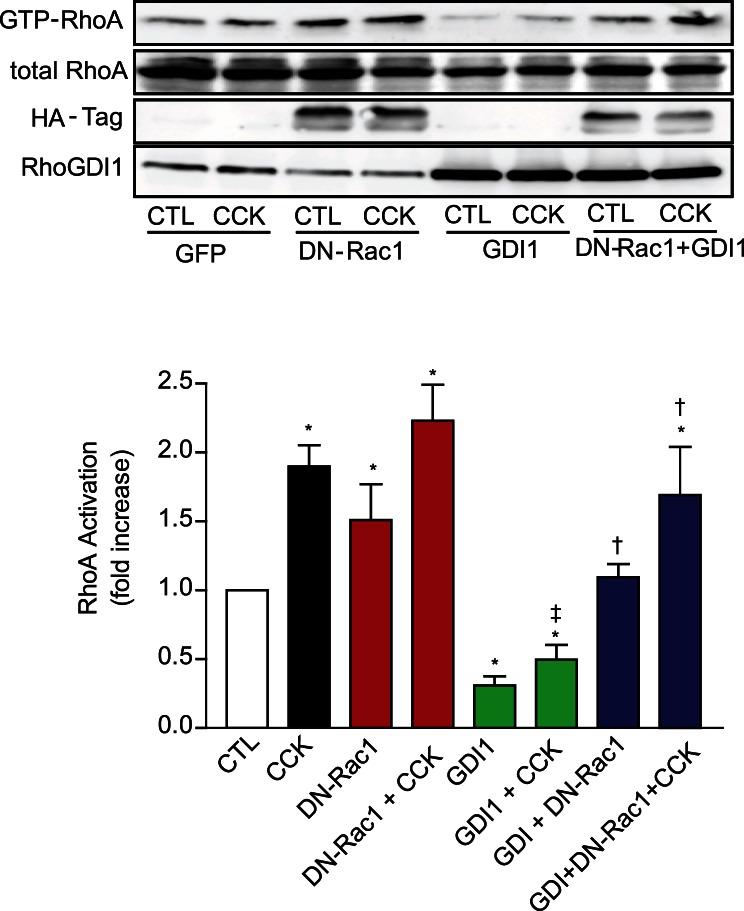
Inactive Rac1 reverses the inhibitory effect of RhoGDI1 on RhoA activation. DN-Rac1, WT-RhoGDI1 or combination of both were expressed in pancreatic acini using adenoviral delivery. Overnight pancreatic acini were stimulated with CCK (300 pM) for 5 min and then RhoA activation was determined by pull-down assay. *Top:* A representative immunoblot for RhoA shows that the expression of DN-Rac1 increases the amount of GTP-RhoA in both non-stimulated and stimulated conditions. The expression of WT-RhoGDI1 inhibits RhoA activation by sequestering inactive RhoA. The co-expression of DN-Rac1 and WT-RhoGDI1 prevents the inhibitory effect of RhoGDI1 on RhoA activation. Comparable expression of DN-Rac1 and WT-RhoGDI1 was analyzed using anti-HA-tag and anti-RhoGDI1 antibodies, respectively. *Bottom:* A quantitative analysis of RhoA activation. Values are means ± SE (n  = 4 experiments). *: p<0.05 vs control, †: p<0.05 vs RhoGDI1 and ‡: p<0.05 vs CCK.

Given Rac1 is also associated with RhoGDI1 we studied whether inactive Rac1 could influence RhoA activation by binding to RhoGDI1. The overexpression of DN-Rac1 increased the amount of GTP-RhoA levels in isolated pancreatic acini in non-stimulated and stimulated conditions **(** **Fig. 9** **).** By contrast, the overexpression of WT-RhoGDI1 inhibited the activation of RhoA by CCK as shown above (**Fig. 8** **and Fig. S4**). The expression of DN-Rac1 prevented the inhibition of WT-RhoGDI by sequestering RhoA because an increase in GTP-RhoA levels was seen upon CCK-stimulation **(** **Fig. 9** **).** These results indicate that inactive Rac1 influences CCK-induced RhoA activation by preventing RhoGDI1 from binding inactive RhoA. Another interesting observation was that the overexpression of DN-Rac1 decreased the expression of RhoGDI1. A further discussion of this observation is beyond the scope of this paper.

### Phosphorylation at Ser96 in RhoGDI1 is Required for CCK-Induced Amylase Secretion

**Figure 10:**
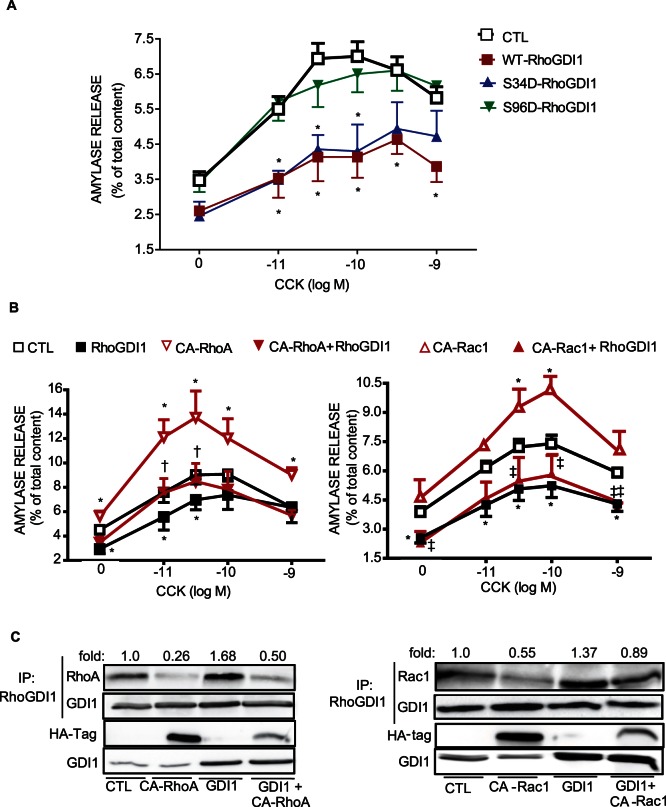
The overexpression of WT-RhoGDI1 or S34D-RhoGDI1 inhibits CCK-induced amylase secretion whereas S96D-RhoGDI1 does not. **A)** Isolated pancreatic acini expressing β-Gal, WT-RhoGDI1 and the phosphomimetic mutants S34D and S96D were stimulated with different concentrations of CCK for 30 min, and amylase release was measured. **RhoGDI1 plays a negative regulatory role on amylase secretion by sequestering inactive RhoA**. **B)** CA-RhoA, CA-Rac1 and WT-RhoGDI1 were co-expressed in pancreatic acini. Isolated pancreatic acini expressing WT-RhoGDI1, CA-RhoA, CA-Rac1 or the combination of both were stimulated with different concentration of CCK for 30 min, and amylase release was measured. The expression of CA-RhoA (left panel) reversed the inhibitory effect of overexpressed WT-RhoGDI1 on amylase secretion, whereas the expression of CA-Rac1 (right panel) does not. Values are means ± SE (n  = 3–4 experiments) of amylase release expressed as a percentage of total. * p<0.05 vs. control acini; † p<0.05 vs. RhoGDI1. **Active RhoA and active Rac1 do not interact with RhoGDI1**. **C)** The association between CA-RhoA or CA-Rac1 with RhoGDI1 was analyzed in total lysates using co-immunoprecipitation. The expression of CA-RhoA, CA-Rac1 and WT-RhoGDI1 was analyzed by Western-blotting using anti-HA-tag and anti-RhoGDI1 antibodies, respectively. A representative immunoblot shows that RhoGDI1 does not form a complex with CA-RhoA or CA-Rac1. Comparable amount of immunoprecitated RhoGDI1 was confirmed by Western-blotting using anti-RhoGDI1 antibody. Data are representative of 4 independent experiments; the dissociation levels were normalized to control  = 1.0 in each assay and expressed as fold change.

The functional relevance of RhoGDI1 and its phosphorylated forms was evaluated in pancreatic acini. As shown in **Fig. 10A**, the overexpression of WT-RhoGDI1, which sequestered both RhoA and Rac1 in the cytosol **(** **Fig. 7** **)**, inhibited CCK-induced amylase secretion from mouse pancreatic acini by 44%. The expression of phosphomimetic mutant S34D-RhoGDI1 (**Fig. 10A**) reduced amylase secretion to the same extent as WT-RhoGDI1. By contrast, the expression of S96D-RhoGDI1, which was not able to sequester either RhoA or Rac1 **(** **Fig. 7B and 7D** **)**, did not affect the response to CCK (**Fig. 10A**). These data indicate that RhoGDI1 is a regulator of amylase secretion and that phosphorylation of RhoGDI1 at Ser96 is required for CCK-induced amylase secretion.

### RhoGDI1 Inhibits CCK-induced Amylase Secretion by Binding Inactive RhoA

Next, we studied whether the inhibitory effect of RhoGDI1 on CCK-induced amylase secretion is by sequestering inactive RhoA, inactive Rac1, or both in the cytosol. For this purpose, a constitutively active mutant of RhoA or Rac1 was expressed in pancreatic acini with or without WT-RhoGDI1. We found that the expression of CA-RhoA or CA-Rac1 enhanced the effect of CCK on amylase secretion as previously shown [16], whereas the expression of WT-RhoGDI1 inhibited the response to CCK **(** **Fig. 10B** **)**. The co-expression with CA-RhoA reversed the inhibition induced by WT-RhoGDI1 **(** **Fig. 10B** **, left)** whereas the co-expression with CA-Rac1 did not (**Fig. 10B** **, right**). Using co-immunoprecipitation, we further confirmed that WT-RhoGDI1 sequestered either inactive RhoA or inactive Rac1 **(** **Fig. 10C** **)** . These findings indicate that RhoGDI1 reduces CCK-induced amylase secretion by sequestering inactive RhoA in the cytosol, and thereby, inhibits RhoA-mediated signaling. The effect of sequestration of Rac1 by RhoGDI1 is not as critical as sequestering of RhoA for regulating amylase secretion.

## Discussion

In the present study, the mechanism by which RhoA translocation is regulated in mouse pancreatic acini upon CCK stimulation was determined. We showed for the first time that PKCα is required for CCK-induced RhoA translocation because: 1) both the broad spectrum PKC inhibitor GF-109203X and the conventional PKC inhibitor Gö6976 reduced CCK-induced RhoA translocation, 2) the phorbol ester PMA induced RhoA translocation, which was inhibited by Gö-6976, 3) the expression of DN-PKCα decreased the response to CCK, and 4), in the presence of PKCα peptide inhibitor, CCK-induced RhoA translocation was essentially inhibited. No further additional conventional PKC isoform is involved in the response to CCK because when pancreatic acini were pre-treated with GF-109203X and DN-PKCα was expressed, there was no further inhibition of RhoA translocation induced by CCK. Gα13 is known to participate in CCK-induced RhoA activation in pancreatic acini [18]. Here, we show that Gα13 is also involved in RhoA translocation because the expression of p115-RGS inhibited the increase in RhoA in the particulate fraction upon CCK stimulation. Although both Gα13 and PKCα participate in the regulation of RhoA translocation, these pathways are not dependent on each other because in conditions where Gα13 is inactive, CCK still induces PKCα translocation.

A recent paper reported the participation of PKCα in alcohol/CCK-evoked pancreatic acinar basolateral secretion [30]. Using PKC peptide inhibitors, the authors demonstrated that PKCα inhibition, but not PKCδ or PKCε inhibition, reduces dramatically the combined response of supramaximal CCK (10 nM) and ethanol (20 mM) on amylase secretion. Of note, the PKCα inhibitors (PKCα myristolated pseudosubstrate inhibitor and the conventional PKC inhibitor Gö6976) do not affect submaximal (50 pM) or maximal concentration of CCK (0.8 nM)-stimulated amylase secretion [30]. These results raise the question of whether membrane translocation of RhoA induced by PKCα is relevant to physiological apical amylase secretion. We suggest that, although Rho GTPases are required for CCK-induced apical amylase secretion, RhoA translocation induced by PKCα is not required for apical amylase secretion. In fact, we consider that PKCα-induced RhoA translocation is involved in basolateral secretion, which is associated with a reduction of apical supramaximal secretion, as well as the formation of basolateral membrane protrusion (blebs). Basolateral exocytosis is a process that occurs at high concentrations of CCK, is associated with acute pancreatitis and involves PKCα activation [30]. Thus, PKCα-induced translocation of RhoA is likely to be part of this event, not only because RhoA translocation occurs at high concentrations of CCK and very rapidly (5 min after stimulation), but also because it requires PKCα activation. The participation of RhoA in the formation of basolateral membrane protrusions (blebs) has been previously shown [16]. Because the direct evidence of RhoA translocation in acinar basolateral membrane protrusions, basolateral secretion, and in the inhibition of apical supramaximal secretion is beyond the scope of the paper, we do not further test this hypothesis.

PKA has been implicated in the regulation of RhoA translocation in some cell types [31], [32], [33]. In migrating cells such as epithelial cells, PKA phosphorylation of RhoA at Ser188 decreases RhoA activation and increases the affinity of RhoA for RhoGDI1, which in turn, induces the extraction of RhoA from the membrane and translocation to the cytosol [31], [34]. Unlike epithelial cells, in pancreatic acini PKA is not required for RhoA translocation. PKA inhibitor did not affect the response to CCK and the stimulants that increase cAMP levels, forskolin and 8-Br-cAMP did not induce RhoA translocation. The lack of an effect of active Gαs on RhoA activation has previously been shown [18], supporting the observation that in pancreatic acini RhoA-mediated signaling is independent of Gαs/cAMP/PKA pathway. Calcium, through activation of PKCα, has regulated the translocation and activation of Rac1 [35]. In our study, we found that CCK induces RhoA translocation through PKCα activation independently of calcium because a calcium chelator, BAPTA-AM, did not modify the response to CCK.

The participation of RhoGDI1 in the regulation of Rho GTPases activity in pancreatic acini and the switch mechanism responsible for the complex dissociation were also studied. First, we found that both RhoGDI1 and RhoGDI3 are expressed in pancreatic acini. Using cell subfractionation and immunohistochemistry, we did not observe changes in the distribution of RhoGDI1 upon CCK stimulation. RhoGDI1 is located in the cytosol and binds cytosolic inactive RhoA and inactive Rac1, but not Cdc42. Of note, the lack of effect of Cdc42 on amylase secretion has been previously shown because the expression of dominant negative Cdc42N17 did not modify the secretory response to CCK [17]. It will be of interest to establish the stiochimetry of RhoGD1/RhoA and RhoGDI1/Rac1. Because the total amount of RhoGDI1 is roughly equivalent to the sum of the levels of the three major Rho proteins: RhoA, Rac1 and Cdc42 [1], the stiochiometry of RhoGDI1/Rac1 and RhoGDI1/RhoA should be close to 1∶1. When the ability of CCK to release RhoA and Rac1 from RhoGDI1 was compared, RhoA was released faster than Rac1. Although a slight decrease in the association between Rac1 and RhoGDI was seen at 5 min, Rac1 was dramatically dissociated at 15 min. These results could be related to the fact that RhoA regulates both early and late phases of apical amylase secretion, whereas Rac1 regulates only the late phase [17]. The release of Rho GTPases from RhoGDI1 was observed at concentrations of CCK higher than 300 pM. The release of Rho GTPases at lower concentrations of CCK can occur but it is difficult to observe using co-immunoprecipitation. We suggest that upon CCK stimulation at lower concentration, a lower amount of Rho GTPases is release from RhoGDI1, and this amount is enough for regulating amylase secretion. It is important to note that CCK-induced amylase secretion is mainly evoked by an increase in calcium levels. At this point, small G proteins play a regulatory role in amylase secretion [26].

CCK stimulation releases RhoA from RhoGDI1 in a PKCα-dependent mechanism because the specific PKCα inhibitor Gö-6976 prevented the dissociation of the complex while the effect of CCK was mimicked by PMA. Either calcium or Gα13 is not required for the complex dissociation. CCK-induced RhoA and Rac1 dissociation from RhoGDI1 was critical for RhoA and Rac1 signaling because the expression of WT-RhoGDI1 inhibited not only CCK-induced RhoA translocation, but also CCK-stimulated RhoA and Rac1 activation. The inhibitory effect of WT-RhoGDI1 on RhoA activation was reversed when inactive Rac1 was overexpressed in pancreatic acini. These results imply a competitive binding of inactive Rho GTPases to RhoGDI1.

We also studied the importance of RhoGDI1 in amylase secretion in response to CCK. RhoGDI1 is implicated in CCK-induced amylase secretion because the expression of WT-RhoGDI1 reduced the response to CCK. By sequestering mostly inactive RhoA, RhoGDI1 exerts its action because the co-expression with CA-RhoA reversed the inhibitory effect of WT-RhoGDI1 on amylase secretion, whereas the co-expression with CA-Rac1 did not. Because all the functional studies were done with the overexpressed protein, it will be of interest to study the effect of CCK on amylase secretion in tissue-specific RhoGDI1 knockout mice. The participation of RhoGDI1 in the regulation of secretion has also been shown in pancreatic β-cells [36], bovine chromaffin cells [37] and mast cells [38].

Two PKCα phosphorylation sites on RhoGDI1 have been identified: in fibroblasts RhoGDI1 has been phosphorylated at Ser34 [5], whereas in endothelial cells RhoGDI1 has been phosphorylated at Ser96 [4]. In both cases the PKCα phosphorylation of RhoGDI1 has selectively increased GTP-RhoA levels, but not GTP-Rac1 or GTP-Cdc42 levels. Moreover, PKCα phosphorylation at Ser96 of RhoGDI1 releases, and thereby activates RhoG, which promotes the activation of Rac1 [39]. Similar to results in endothelial cells [4], [39], in pancreatic acini, phosphorylation of RhoGDI1 at Ser96 is required for the dissociation of RhoA-RhoGDI1 complex, and thereby RhoA-mediated signaling. By mutational analysis we found that CCK stimulation failed to induce RhoA activation in pancreatic acini expressing the phosphodefective mutant S34A-RhoGDI1, as well as the phosphomimetic mutant S34D-RhoGDI1. By contrast, the expression of phosphomimetic mutant S96D-RhoGDI1 reduced the affinity of RhoGDI1 for RhoA, and thereby enabled the response to CCK, whereas the expression of the phosphodefective mutant S96A-RhoGDI1 conserved the inhibitory activity of RhoGDI1 toward RhoA, inhibiting the response to CCK. Unlike a previous finding [4], the phosphorylation at Ser96 was also required for releasing Rac1 from RhoGDI1 and, thereby, CCK-induced Rac1 activation. RhoGDI1 is composed of a flexible 69-amino-acid N-terminus and an immunoglobulin-like, folded, 135-amino acid C-terminus. The N-terminus contains the nucleotide binding site, and the C-terminus contains a hydrophobic pocket that binds the isoprenyl group of each member of the Rho family. This finding further supports previous studies which show that the dissociation of Rho GTPases from RhoGDI1 requires the dissociation between the folded C-terminal domain of RhoGDI1 and the isoprenyl group of Rho GTPases [4], [8], [10].

**Figure 11:**
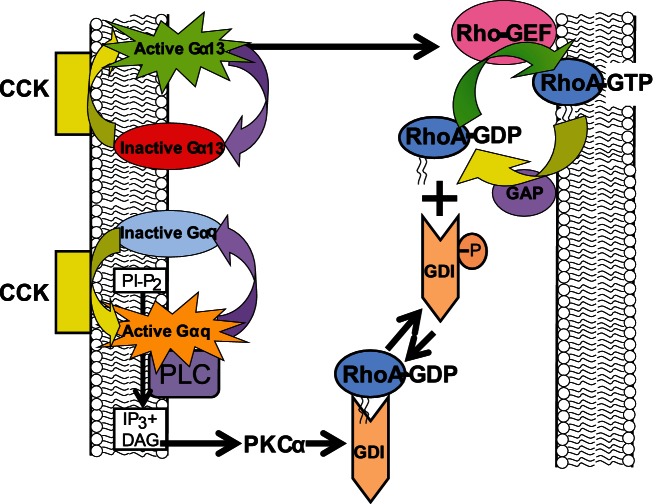
A schematic presentation describing the different signaling components involved in the effect of CCK on RhoA signaling. Inactive RhoA is localized in the cytosol complexed to RhoGDI1, which masks the geranylgeranylated group. Upon CCK stimulation, two pathways are activated: Gα13 pathway and PKCα pathway. RhoGDI1 is phosphorylated at Ser96 by PKCα, and thereby, releases inactive RhoA. Once inactive RhoA is dissociated, inactive RhoA is able to translocate to membranes and be activated by RhoGEF, in a mechanism which dependent on active Gα13. Finally, GAP inactivates RhoA, which is able to associate with cytosolic RhoGDI1.

Based on the results reported in this manuscript, we suggest the following mechanism: inactive RhoA is localized in the cytosol complexed to RhoGDI1, which masks the geranylgeranylated group. Upon CCK stimulation, two pathways are activated: Gα13 pathway and Gαq pathway, which involves PKCα activation. RhoGDI1 is phosphorylated by PKCα, and thereby, releases inactive RhoA, which is able to translocate to membranes and be activated by RhoGEF, in a mechanism dependent on active Gα13 [18]. Finally, GAP inactivates RhoA, which is able to associate with cytosolic RhoGDI1 **(** **Fig. 11** **).** The Gα13/RhoA pathway promotes actin cytoskeleton disassembly independently of calcium [26], [40]. These findings support previous data showing that the effect of Gα13 on amylase secretion is calcium-independent because the expression of p115-RGS did not modify CCK-induced calcium mobilization [18].

In conclusion, inactive RhoA and inactive Rac1 are localized in the cytosol bound to RhoGDI1. Upon CCK stimulation are both RhoA and Rac1 released from RhoGDI1 to facilitate their activation and to mediate apical amylase secretion. Both Gα13 and PKCα, independently, contribute to this event. PKCα is involved in the dissociation of RhoA from RhoGDI1. Once RhoA is dissociated, RhoA is able to translocate to membranes and be activated by RhoGEF, in a Gα13-dependent mechanism.

## Supporting Information

Figure S1 **An increase in cAMP levels does not induce RhoA translocation.** **A)** Isolated pancreatic acini were stimulated for 5 min with 100 pM CCK, 20 µM forskolin (FSK) and 100 µM 8-Br-cAMP, lysed and separated into cytosolic and particulate fractions. None of cAMP pathway stimulators induced an increase in RhoA levels in the particulate fraction. **CCK-induced RhoA translocation is calcium-independent.** **B)** Isolated pancreatic acini were pretreated with the calcium chelator BAPTA-AM (25 µM) and then stimulated for 5 min with 100 pM CCK. Calcium chelation did not affect the response to CCK. *Top:* A representative immunoblot for RhoA. *Bottom*: A quantitative analysis of RhoA translocation. Values are means ± SE (n  = 3 experiments). * p<0.05 vs. control (CTL).
(EPS)

Figure S2 **Only RhoGDI1 and RhoGDI3 are expressed in mouse pancreatic acini.** **A)** RhoGDI isoforms expression was assessed in mouse pancreatic acini using RT-PCR with brain, heart, lung and kidney used as positive controls. Both RhoGDI1 and RhoGDI3 mRNA expression were observed in mouse pancreatic acini and yielded products of the expected size (382 bp and 344 bp, respectively) while the PCR product for RhoGDI2 (323 bp) was observed in brain, heart, lung, weakly expressed in the whole pancreas, but not in pancreatic acini. **RhoGDI1 localizes in the cytosol in both non-stimulated and stimulated conditions.** **B)** Isolated pancreatic acini were treated with the phorbol ester PMA, the calcium ionophore A23187 and CCK for 5 min, lysed and separated into cytosolic and particulate fractions. A representative immunoblot for RhoGDI1 shows that RhoGDI1 is located in the cytosol and its localization is not modified upon stimulation. **Immunolocalization of RhoGDI1 in pancreatic acini.** **C)** Isolated pancreatic acini were stimulated with **(B)** or without **(A)** 300 pM CCK for 15 min. Immunohistochemistry was used to localize RhoGDI1 (red). Nuclei were stained with 4,6-diamino-2-phenylindole (DAPI) (blue). RhoGDI1 was diffusely localized in the cytosol. No change in the localization of RhoGDI1 is observed upon CCK stimulation.
(EPS)

Figure S3 **RhoGDI1 interacts with inactive RhoA and Rac1.** Pancreatic acini expressing β-Gal (control), HA-CA-RhoA, HA-DN-RhoA, HA-CA-Rac1 and HA-DN-Rac1 were subjected to HA-immunoprecipitation. The immunoprecipitated HA-tagged proteins were specifically eluted with HA peptide and elutes were subjected to Western-blotting for RhoGDI1. A representative immunoblot for RhoGDI1 shows that only HA-DN-RhoA and HA-DN-Rac1 are able to interact with RhoGDI1. Equivalent loading was confirmed by Western-blotting using anti-HA antibody. Comparable expression of HA-fusion proteins was confirmed by Western-blotting using anti-HA antibody. CTL: Control (n: 3 experiments).
(EPS)

Figure S4 **The expression of WT-RhoGDI1 and its S34A and S96A mutants inhibit CCK-stimulated RhoA activation.** Wild-type (WT) and the phosphodefective mutants S34A-RhoGDI1 and S96A-RhoGDI1 were expressed in pancreatic acini using adenoviral delivery. RhoA activation was studied using RhoA pull-down assay. Comparable expression of WT-RhoGDI1 and its mutants was analyzed by Western-blotting using an anti-RhoGDI1 antibody. *Top:* A representative immunoblot for RhoA shows that WT-RhoGDI1, as well as its phosphodefective mutants inhibit CCK-stimulated RhoA activation. *Bottom*: A quantitative analysis of RhoA activation. Values are means ± SE (n: 3 experiments). **: p<0.01 vs control (CTL) and †: p<0.05 vs CCK.
(EPS)

Figure S5 **Phosphorylation of Ser96 in RhoGDI1 is required for CCK-induced RhoA translocation.** Wild-type (WT) and the phosphodefective mutants S34A-RhoGDI1 and S96A-RhoGDI1, as well as phosphomimetic mutants S34D-RhoGDI1 and S96D-RhoGDI1 were expressed in pancreatic acini using adenoviral delivery. RhoA translocation was studied using differential centrifugation. Comparable expression of the WT-RhoGDI1, as well as mutants was analyzed by Western-blotting using an anti-RhoGDI1 antibody. A representative immunoblot shows that in acini expressing the WT-RhoGDI1, as well as the phosphodefective mutants S34A-RhoGDI1 and S96A-RhoGDI1, CCK-induced RhoA translocation was abolished. Only the expression of S96D-RhoGDI1 facilitates CCK-induced RhoA translocation (Representative of 4 experiments). CTL: Control.
(EPS)
